# Elevated serum anti-flagellin antibodies implicate subclinical bowel inflammation in ankylosing spondylitis: an observational study

**DOI:** 10.1186/ar4350

**Published:** 2013-10-26

**Authors:** Dinny Wallis, Arundip Asaduzzaman, Michael Weisman, Nigil Haroon, Ammepa Anton, Dermot McGovern, Stephan Targan, Robert Inman

**Affiliations:** 1 Toronto Western Hospital, University of Toronto, 399 Bathurst St, Toronto M5T 2S8, ON, Canada; 2 Cedars-Sinai Medical Center, Los Angeles, CA, USA

## Abstract

**Introduction:**

Ankylosing spondylitis (AS) and inflammatory bowel disease (IBD) share genetic and clinical features. IBD is associated with the presence of antibodies to a variety of commensal microorganisms including anti-*Saccharomyces cerevesiae* antibodies (ASCA), antineutrophil cytoplasmic antibodies (ANCA), anti-I2 (associated with anti-*Pseudomonas* activity), anti-*Eschericia coli* outer membrane porin C (anti-OmpC) and anti-flagellin antibodies (anti-CBir1). Subclinical intestinal inflammation may be present in up to 65% of patients with AS. This study evaluated the presence of antimicrobial antibodies in patients with AS alone, patients with AS and concomitant IBD (AS-IBD) and a control group of patients with mechanical back pain (MBP).

**Methods:**

Sera were tested by ELISA for ASCA IgG and IgA, anti-OmpC, anti-CBir1 and ANCA in 76 patients with AS alone, 77 patients with AS-IBD and 48 patients with MBP. Antibody positivity rates, median quantitative antibody levels and the proportion of patients with antibody levels in the 4^th^ quartile of a normal distribution were compared between the three groups of patients.

**Results:**

Patients with AS alone demonstrated higher anti-CBir1 antibody positivity rates and median antibody levels than MBP patients. Anti-CBir1 positivity in AS was associated with elevation of acute phase reactants. AS-IBD patients demonstrated elevated responses when compared to AS alone for ASCA, anti-OmpC and anti-CBir1. Quartile analysis confirmed the findings.

**Conclusions:**

These data suggest that adaptive immune responses to microbial antigens occur in AS patients without clinical IBD and support the theory of mucosal dysregulation as a mechanism underlying the pathophysiology of AS.

## Introduction

Ankylosing spondylitis (AS) is a chronic inflammatory arthritis characterized by inflammation of the joints of the spine, tendons and entheses. An association between AS and inflammatory bowel disease (IBD) has been recognized for many years. Evidence of intestinal inflammation, which may be subclinical, is present in up to 65% of patients with spondyloarthritis (SpA) [1]. In axial spondyloarthritis, subclinical gut inflammation has been shown to be independently associated with male sex, high disease activity, restricted spinal mobility and shorter symptom duration [2]. There is evidence to support a common genetic component for AS and IBD, as evidenced by a study of families of AS probands in Iceland [3]. Further work has shown that a single nucleotide polymorphism (SNP) in the IL-23R) gene on chromosome 1p31 is associated with Crohn’s disease (CD) and psoriasis [4]. Analysis of three distinct AS populations in Canada has demonstrated a disease association with the IL-23 receptor (IL-23R) locus and implicates the same polymorphism associated with IBD and psoriasis [5]. Recent genome-wide association studies have further highlighted commonalities in genetic susceptibility to IBD and AS [6].

IBD is associated with a variety of serological antibodies, which suggests loss of tolerance to a subset of commensal microorganisms [7]. These include: (i) anti-*Saccharomyces cerevesiae* antibodies (ASCA) directed against a cell wall polysaccharide of the yeast; (ii) antineutrophil cytoplasmic antibodies (pANCA); (iii) anti-I2 (associated with anti-*Pseudomonas* activity) particularly in Crohn’s disease (CD); (iv) anti-*Eschericia coli* outer membrane porin C (anti-OmpC) and (v) anti-flagellin (anti-CBir1) antibodies. Circulating antibodies may be useful in distinguishing patients with IBD from healthy controls and from other gastrointestinal disorders. For example, sensitivity of ASCA for IBD ranges from 31 to 45% and specificity from 90 to 100% [8]. The role of circulating antibodies in the pathogenesis of IBD is not understood but it is generally accepted that they reflect an aberrant immune response rather than the recognition of specific or pathogenic bacteria.

The presence of these antibodies in AS patients has been investigated in a pilot study conducted in the USA [9]. There was no difference in positivity rates between AS and control groups with the established IBD values of antibodies. When antibody levels were distributed into quartiles, AS patients were more likely than controls to have a quartile score of 4 (upmost quartile) for anti-I2, ASCA immunoglobulin (Ig) G and total ASCA. To further define the relationship of these antibodies with AS and IBD, we studied antimicrobial antibody reactivity in a cohort of AS patients with and without concomitant IBD, compared to mechanical back pain (MBP) controls.

## Methods

### Patients

Patients attending the Toronto Western Hospital Spondylitis Clinic are invited to be registered in the SpA database. All patients provide written consent to participate in the cohort and the project has been approved by the Research Ethics Board of Toronto University Health Network in accordance with the Helsinki Declaration. Clinical, laboratory and radiological data are collected according to a standardized protocol with concomitant serum banking. Patients are individually examined by a rheumatologist annually, which includes a comprehensive clinical examination and a full medical history including details of gastrointestinal and other extra-articular symptoms. Sera are frozen and stored in micro-aliquots at -80C with no freeze-thaw cycles. All patients diagnosed with both AS (according to modified New York Criteria) and IBD (confirmed by a gastroenterologist) and for whom serum was available, were identified by review of the database and clinical records. Patients were matched for age and disease duration (both within 2 years) to a group of AS patients without IBD. A control group was included, which consisted of mechanical back pain (MBP) patients whose serum had previously been stored in the same manner. All MBP patients underwent a clinical assessment, spinal radiography and laboratory investigations to rule out SpA. Clinical characteristics of the patients at the time of serum sampling were extracted from the database and clinical records.

### Laboratory methods

Serum samples from the earliest patient visit available were tested by ELISA for ASCA IgG and IgA, anti-OmpC, anti-CBir1 and ANCA at Cedars-Sinai Medical Centre (Los Angeles, CA, USA). Basic laboratory methods have been described elsewhere [7,10]. The results of each assay are expressed in ELISA units and reflect antibody levels at 1/100 dilution.

### Statistical methods

Data were analyzed using GraphPad Prism 6 software. Disease characteristics were compared using Fisher’s exact test with two-tailed *P*-values and the Mann-Whitney test. Chi-squared testing was performed to compare the antibody positivity rates in AS-IBD patients, patients with AS alone and patients with MBP. The reference values usually applied to IBD were used [9]. Pairwise comparisons were then performed using Fisher’s exact test with correction for multiple comparisons using the Benjamini-Hochberg method. Median quantitative antibody levels were compared using one-way analysis of variance (ANOVA) with Dunn’s test for multiple comparisons. Finally, quantitative antibody levels were log-transformed and distributed into quartiles. A quartile score was allocated on the basis of the quartile (<25% = 1; 25 to 50% = 2; 51 to 75% = 3; >75% = 4). Chi-squared testing was performed to compare the proportion of patients with a quartile score of 4 in AS-IBD, AS and MBP. Pairwise comparisons were repeated using Fisher’s exact test.

## Results

Serum samples studied were from the following: 77 AS-IBD patients (of whom forty-eight had CD, twenty-eight had ulcerative colitis (UC) and one had indeterminate colitis), 76 AS patients and 48 MBP patients. Clinical characteristics of the study population at the time of serum sampling are shown in Table 1. As expected, AS-IBD patients had lower rates of non-steroidal anti-inflammatory drug (NSAID) use and higher rates of disease-modifying anti-rheumatic drug (DMARD) and steroid use. Human leukocyte antigen (HLA)-B27 positive rates were lower in AS-IBD patients than AS alone patients. Biologic use was similar between AS and AS-IBD groups with approximately one quarter of all patients being prescribed a biologic.

**Table 1:** Characteristics of the study population at time of serum sampling

	**AS (n = 76)**	**IBD-AS (n = 77)**	***P*-value**
Age, y	39.5 (28.5 to 50.5)	39.0 (31.0 to 50.0)	ns
Disease duration, y	5.0 (1.0 to 11.8)	6.0 (1.5 to 11.5)	ns
Male, %	86	78	ns
HLA-B27 positive, %	82	59	0.0033
BASDAI, NRS, 0 to 10	4.2 (2.5 to 6.8)	3.3 (2.4 to 6.7)	ns
BASFI, NRS, 0 to 10)	4.1 (1.4 to 6.4)	3.2 (0.7 to 6.1)	ns
Biologic current, %	24	30	ns
DMARD current, %	11	35	0.0004
Steroid current, %	3	14	0.0173
NSAID current, %	63	39	0.0036
History of uveitis, %	21	31	ns
History of psoriasis, %	7	7	ns
Current smoker, %	28	30	ns
ESR, mm/h	15.7 (3.0 to 25.2)	15.3 (5.3 to 22.5)	ns
CRP, mg/L	6.5 (3.0 to 15.5)	9.0 (3.0 to 19.0)	0.0532

Values are median (IQR) unless stated otherwise. For comparison: mechanical back pain patients (n=48): age 43.0 (34.8 to 53.0) y, 56.3% male, ESR 4.0 (2.0 to 7.5) mm/h, CRP 3.0 (3.0 to 3.0) mg/L. HLA, human leukocyte antigen; BASDAI, Bath Ankylosing Spondylitis Disease Activity Index; BASFI, Bath Ankylosing Spondylitis Functional Index; NRS, numerical rating scale; DMARD, disease modifying anti-rheumatic drug; NSAID, non-steroidal anti-inflammatory drug; CRP, C-reactive protein; ESR, erythrocyte sedimentation rate; ns, not significant.

### Positivity rates of serological tests

**Figure 1:**
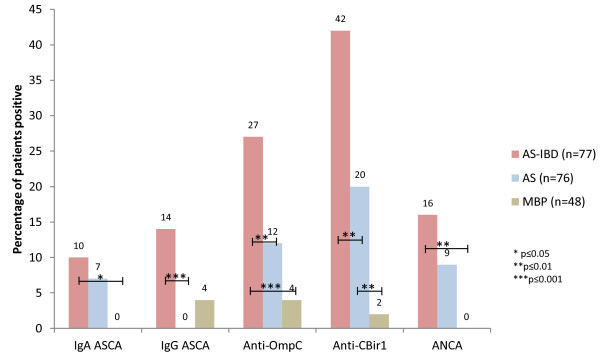
**Positivity rates of all antibodies.** AS-IBD, ankylosing spondylitis-inflammatory bowel disease; AS, ankylosing spondylitis; MBP, mechanical back pain; Ig, immunoglobulin; ASCA, anti-Saccharomyces cerevesiae antibody; Anti-OmpC, anti-outer membrane porin C; Anti-CBir-1, anti-flagellin; ANCA, anti-neutrophil cytoplasmic antibody.

Significant differences were identified in the positivity rates for all antibodies tested between AS, AS-IBD and MBP patients. Results of pairwise comparisons are shown in Figure 1. AS-IBD patients demonstrated higher positivity rates of ASCA IgG, anti-OmpC and anti-CBir1 than patients with AS alone. Within the AS-IBD group, CD patients were more likely than UC patients to be positive for anti-CBir1 (47.9% versus 21.5%, *P* = 0.028) and anti-OmpC (31.3% versus 7.1%, *P* = 0.021). A trend was seen for higher IgG ASCA positivity rates in CD than UC (18.8% versus 3.6%, *P* = 0.082). AS patients had a higher positivity rate for anti-CBir1 antibody and ANCA than MBP patients. Positivity rates for IgA ASCA, anti-OmpC, anti-CBir1 and ANCA were higher in AS-IBD than MBP.

### Quantitative antibody levels

**Figure 2:**
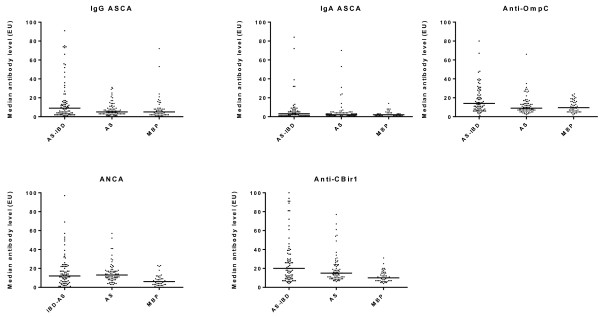
**Median quantitative antibody levels in ankylosing spondylitis-inflammatory bowel disease, ankylosing spondylitis and mechanical back pain.** Outliers are not shown. AS, ankylosing spondylitis; AS-IBD, ankylosing spondylitis and inflammatory bowel disease; MBP, mechanical back pain; Ig, immunoglobulin; ASCA, anti-*Saccharomyces cerevesiae* antibody; Anti-ompC, anti-outer membrane porin C; Anti-CBir1, anti-flagellin.

Significant differences in the median quantitative antibody levels between the three patient groups were identified for anti-OmpC, anti-CBir1 and ANCA. Pairwise comparisons were performed to identify which patient groups differed (Figure 2). Anti-OmpC levels were higher in AS-IBD when compared to both AS and MBP, but did not differ between AS and MBP. Anti-CBir1 antibody and ANCA levels were similar in AS-IBD and AS and were significantly elevated in both these groups when compared to MBP.

The antibody results were distributed into quartiles. A greater proportion of AS-IBD patients had a quartile score of 4 (highest quartile) than AS patients, for IgA ASCA (36.4% versus 21.1%, *P* = 0.049), IgG ASCA (35.1% versus 17.9%, *P* = 0.016), anti-OmpC (37.7% versus 18.4%, *P* = 0.001) and anti-CBir1 (42.9% versus 19.7%, *P* = 0.003). Patients with AS were more likely to have a quartile score of 4 than MBP patients for anti-CBir1 (19.7% versus 4.2%, *P* = 0.016) and ANCA (26.3% versus 8.3%, *P* = 0.019). AS-IBD patients were more likely to have a quartile score of 4 than MBP patients for all antibodies except IgG ASCA, where the difference did not reach significance.

### Clinical characteristics of anti-CBir1-positive patients

Differences in clinical characteristics between anti-CBir1-positive and anti-CBir1-negative patients were investigated. When AS and AS-IBD patients were combined, a lower rate of biologics use was observed in anti-CBir1-positive patients (13.6%) compared to anti-CBir1-negative patients (32.1%, *P* = 0.0257). Acute phase reactants were significantly elevated in anti-CBir1-positive patients compared to anti-CBir1-negative patients: median (IQR) C-reactive protein (CRP) 11.5 (5.3 to 20.5) mg/L versus 6.0 (3.0 to 15.5) mg/L (*P* = 0.006), and median (IQR) ESR 19.0 (8.5 to 32.0) mm/h versus 8.0 (3.0 to 18.0) mm/h (*P* <0.001). When AS and AS-IBD patients were analyzed separately, a trend for lower rate of biologic use in anti-CBir1-positive patients was seen in both groups but the difference was not significant. Among patients with AS alone, acute phase reactants remained significantly elevated in anti-CBir1-positive patients compared to anti-CBir1-negative patients (median (IQR) CRP 14.0 (7.0 to 19.0) mg/L versus 3.0 (3.0 to 15.0) mg/L, *P* = 0.033); median ESR (IQR) 26.0 (10.0 to 33.0) mm/h versus 5.0 (3.0 to 20.0) mm/h). No significant difference was seen in acute-phase reactants between anti-CBir 1 positive and negative patients within the AS-IBD group.

## Discussion

The results of this study confirm an elevated serum antibody profile with respect to anti-CBir1 and ANCA in AS patients without clinical IBD, suggestive of mucosal dysregulation in these patients. The level of reactivity for certain antibodies is greater in patients with coexisting IBD than in AS patients alone. The prevalence of anti-CBir1 antibodies in our CD patients (47.9%) was comparable to previously reported rates in CD (50 to 56%) [8]. Quartile analysis confirmed the findings and the only additional difference identified was an elevation of IgG ASCA in AS-IBD compared to AS. It is possible that loss of tolerance to microbial antigens is one mechanism that contributes to subclinical bowel inflammation in AS patients without IBD, and that the magnitude of the antibody response determines the degree of mucosal dysregulation and presence or absence of clinical IBD.

To our knowledge, this is the first study to demonstrate elevated antibody responses in AS compared to MBP using the reference values usually applied to IBD. An earlier study of 80 AS patients and 80 controls free of rheumatic disease [9] did not find any difference in positivity rates for IgG ASCA, IgA ASCA, anti-OmpC, anti-CBir1 or ANCA between the two groups, although higher quantitative antibody quartile scores for ASCA IgG and ASCA IgA were reported in AS compared to controls. Another study investigating ASCA in SpA found that ASCA IgA, but not IgG, levels were higher in AS and undifferentiated SpA than in healthy controls but our work has not confirmed these findings [11]. There was no difference in ASCA levels between SpA patients with and without bowel inflammation. A further study reporting the prevalence of pANCA, ASCA IgA, ASCA IgG and anti-OmpC antibodies in 52 AS patients without IBD found the prevalence of all antibodies to be higher than in our study at 21%, 19%, 8% and 19% respectively [12].

The presence of certain antibodies in IBD has clinical and prognostic significance. In CD, ASCA positivity has been associated with younger age of onset and small bowel involvement [13] whereas pANCA is associated with UC-like features [14]. It is thought that presence of pANCA in IBD may represent cross-reactivity with enteric bacterial antigens [15]. Anti-CBir1 antibodies have been detected in approximately 50% of CD patients and reactivity is associated with fibrostenosing disease and complicated small bowel CD [16]. The presence of anti-CBir1 in AS patients without bowel symptoms may be an indicator of subclinical bowel inflammation or predictor of future IBD. Our finding of significantly elevated acute-phase reactants in anti-CBir1-positive AS patients without IBD would support the theory of subclinical bowel inflammation in AS.

It has been postulated that antibody reactivity in CD may be genetically determined. Genetic variants of the CARD15/NOD2 gene have been associated with CD and the quantitative antibody response for anti-CBir1 has been demonstrated to be elevated in patients with CD carrying at least one Nucleotide-binding oligomerization domain-containing protein 2 (NOD2) variant, although there was no association with the presence of antibody reactivity [10]. Flagellin, a molecular component of bacterial surfaces, interacts with Toll-like receptor 5 (TLR5) and thereby stimulates the production of proinflammatory cytokines. In CD, it has recently been shown that *TLR5-stop* mutation abrogates development of anti-CBir1 in a dominant-negative fashion and in the same study another CD susceptibility gene, *IRGM*, was associated with increased anti-CBir1 seropositivity [17].

There are a number of limitations to the present study. The sample size is relatively small and larger studies are required to confirm the findings. It is possible that drug therapy in our patients influenced antibody reactivity. The earliest serum sample available was used for analysis and a significant number of patients were on biologic therapy at this time, with similar proportions of patients in the AS-IBD and AS groups using a biologic drug. We chose not to exclude these patients since it is has been determined that antibody responses do not change over time or as a result of drug therapy, including biologic therapy. A number of studies have demonstrated that the ASCA, anti-OmpC, anti-I2 and anti-CBir1 antibodies are not altered by treatment with glucocorticoids, mesalazine or infliximab [6]. Our finding that biologic use was associated with anti-CBir1 negativity is unexplained. Patients selected for biologic therapy in AS generally have more severe disease. Further work is required to confirm whether biologic therapy affects serological profiles and how this may be related to underlying disease severity or control of disease activity. The current study did not investigate the relationship between antibody reactivity and objective evidence of subclinical bowel inflammation, for example through the measurement of fecal calprotectin or ileocolonoscopy. Future studies could address this question, while acknowledging undertaking colonoscopy in asymptomatic patients may pose logistical hurdles for such studies. It would also be interesting to measure antibody levels in patients with non-radiographic axial spondyloarthritis, with or without IBD, to investigate whether the presence of antibodies predicts development to radiographic AS, or whether the presence of these antibodies in AS alone predicts development of clinical gut inflammation.

## Conclusions

Patients with AS alone demonstrated higher anti-CBir1 antibody positivity rates and median antibody levels than MBP patients. Anti-CBir1 positivity in AS was associated with elevation of acute-phase reactants. These data suggest that adaptive immune responses to microbial antigens occur in AS patients without clinical IBD and support the theory of mucosal dysregulation as a mechanism underlying the pathophysiology of AS.

## Abbreviations

ANCA: Anti-neutrophil cytoplasmic antibody; ANOVA: Analysis of variance; anti-CBir1: anti-flagellin; anti-OmpC: Anti-outer membrane porin C; AS: Ankylosing spondylitis; ASCA: Anti-*Saccharomyces cerevesiae* antibody; CARD15: Caspase recruitment domain-containing protein 15; CD: Crohn’s disease; CRP: C-reactive protein; DMARD: Disease-modifying anti-rheumatic drug; ELISA: Enzyme linked immunosorbant assay; IBD: Inflammatory bowel disease; Ig: Immunoglobulin; IL-23R: Interleukin 23 receptor; MBP: Mechanical back pain; NOD2: Nucleotide-binding oligomerization domain-containing protein 2; NSAID: Non-steroidal anti-inflammatory drug; pANCA: Antineutrophil cytoplasmic antibodies; SNP: Single nucleotide polymorphism; SpA: Spondyloarthropathy; UC: Ulcerative colitis.

## Competing interests

The authors declare that they have no competing interest.

## Authors’ contributions

DW performed the statistical analysis and drafted the manuscript. AAs and AAn assisted with data analysis. NH and RI contributed to study design and interpretation of results. MW, ST and DM approved the final manuscript for publication. All authors read and approved the final manuscript.
